# Draft genome sequence of naphthalene-degrading marine *Rhodococcus ruber* PCTR3

**DOI:** 10.1128/mra.00003-26

**Published:** 2026-06-18

**Authors:** Tripti Raghavendra, Sarita G. Bhat

**Affiliations:** 1 Department of Biotechnology, Cochin University of Science and Technology, South Kalamassery, Cochin, India; University of Southern California, Los Angeles, California, USA

**Keywords:** marine microbiology, bioremediation, biosurfactants

## Abstract

We report the genome sequence of a marine *Rhodococcus ruber* PCTR3 consisting of 5,754,741 bp (5,306 genes and GC: 70.34%), isolated from sediment from Cochin Port, India. This strain degraded 1,000 mg/L naphthalene within 72 h, and functional annotation indicated multiple xenobiotic degradation enzymes and production of important secondary metabolites.

## ANNOUNCEMENT

**Fig 1:**
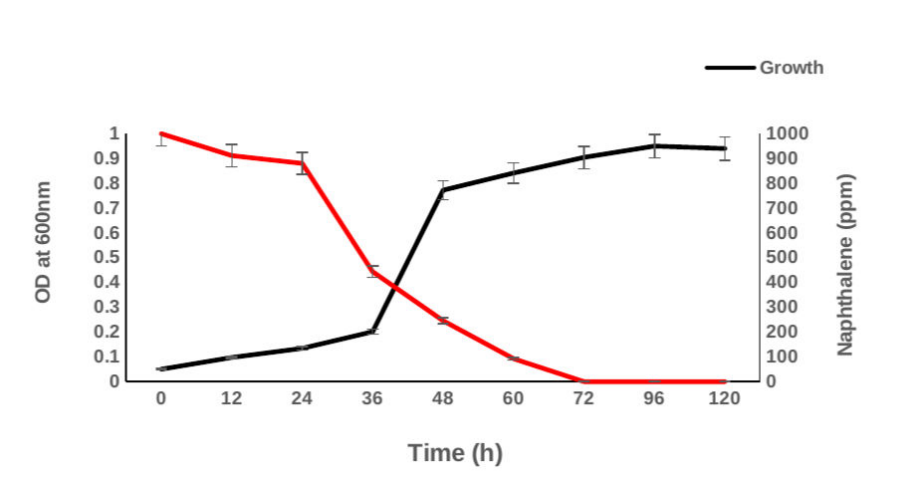
Growth profile and naphthalene degradation (1,000 mg/L) by *R. ruber* PCTR3 in BH broth at 37°C and 150 rpm.

*Rhodococcus ruber* PCTR3 was isolated from a bacterial consortium, PCTRN1, which was developed by an enrichment method in Bushnell Haas broth containing naphthalene as the sole carbon source, using the sediment from Port Cochin, India (9°57′53.2″ N, 76°15′46.1″ E) (1). *R. ruber* PCTR3 was isolated by pure culture techniques using nutrient agar and identified by 16S PCR and BLAST to NCBI nt as described previously (accession no. PV000746) (1). The strain degraded 1,000 mg/L naphthalene (analyzed by GC) within 72 h in BHB medium during the log and early stationary phase (Fig. 1), which was the highest among the five different bacterial strains isolated from PCTRN1 and equivalent to that of the complete consortium (1); hence, *R. ruber* PCTR3 was subjected to whole-genome sequencing (WGS) for further functional annotation.

For WGS, *R. ruber* PCTR3 was grown and maintained in nutrient broth for bacterial culture at 37°C under aeration for 72 h. Genomic DNA was extracted from the pure culture using QIAamp DNA Mini Kit (QIAGEN, Germany) as per the manufacturer’s instruction. Furthermore, the quality and quantity of the extracted DNA were confirmed according to the published article (2). The QC-passed DNA sample was processed for Illumina-compatible WGS library preparation using NEBNext Ultra II FS DNA Library Prep Kit as per the manufacturer’s instructions. Finally, the quality-passed WGS library was sequenced on Illumina NextSeq 2000 platform (2 × 150 bp chemistry).

The raw WGS read quality was assessed using FastQC v0.11.9 (3), and pre-processing of raw reads, which includes removal of adapter sequences and low-quality bases, was performed using the Trimmomatic v0.39 tool (LEADING:5 TRAILING:5 SLIDINGWINDOW:4:20 MINLEN:50 TOPHRED33) (4). The *de novo* whole-genome assembly was carried out using Unicycler v0.5.0 (5) with default parameters, and assembly completeness was confirmed by BUSCO v4 (database: bacteria_odb10) (6). Furthermore, assembly species were confirmed using homology-based approach (BlastN) (7) and TYGS server analysis (8).

In total, 6,613,256 paired-end (PE) raw reads were obtained from the Illumina sequencer. After pre-processing, 4,023,690 good-quality PE reads (*Q* value >30 and coverage: ~200×) were retained. The genome assembly resulted in a draft genome assembly with the size of 5,754,741 bp (N50: 256,262 bp and GC: 70.34%) in 70 scaffolds, with a BUSCO genome completeness score of 99.2%. The homology analysis (against the NCBI-NR database with an *E* value of 0), as well as the TYGS analysis, confirmed that the assembled genome is closely related to the species *Rhodococcus ruber* (accession no. NZ_LRRL00000000).

Gene prediction using NCBI-PGAP (9) resulted in 5,306 genes (tRNA: 53, rRNA: 3, ncRNA: 3, and protein coding: 5,247). Among the predicted protein-coding genes, 5,136, 4,210, and 2,160 genes were functionally annotated using UniProt, COG, and KEGG databases, respectively (10–13). Notably, 137 enzymes related to xenobiotic degradation were annotated by KEGG, of which 93 were specific to hydrocarbon degradation pathways. Various enzymes were annotated for the production of secondary metabolites, including biosurfactants, antibacterial agents, and precursors for value-added products. Overall, this genomic study and wet-lab inferences indicate the possibility that this strain may be a potential bioremediation agent, similar to other *Rhodococcus* spp. ( 14–16).

## Data Availability

The raw sequencing reads have been deposited in the NCBI Sequence Read Archive and are publicly available under accession number SRR36544808. The GenBank accession number for the assembled genome is JBTZCV000000000. The BioProject accession number for this project is PRJNA1392063, and the BioSample accession number is SAMN54242482. The functional annotation data, i.e., Uniprot, KEGG, and COG data, have been deposited in FigShare and are publicly available under https://doi.org/10.6084/m9.figshare.32323497, https://doi.org/10.6084/m9.figshare.32323488, and https://doi.org/10.6084/m9.figshare.32229156, respectively.
